# Socio-demographic and behavioural profile of women in polygamous relationships in South Africa: a retrospective analysis of the 2002 population-based household survey data

**DOI:** 10.1186/s12905-018-0626-9

**Published:** 2018-08-02

**Authors:** Musawenkosi L. H. Mabaso, Nthabiseng F. Malope, Leickness C. Simbayi

**Affiliations:** 1 HAST, Human Sciences Research Council, The Atrium, 5th Floor, 430 Peter Mokaba Street, Berea, Durban, South Africa; 2 Office of the Deputy CEO for Research, Human Sciences Research Council, 116 – 118 Merchant House Building, Buitengracht Street, Cape Town, 8001 South Africa; 3 Department of Psychiatry & Mental Health, University of Cape Town, Cape Town, South Africa

**Keywords:** Polygamy, Marriage, Women, Socio-demographic, Behaviour, South Africa

## Abstract

**Background:**

The prevalence and effect of polygamous relationships may have serious reproductive and /or health consequences for women. In South Africa, unlike in other sub-Saharan countries, no nationwide survey has investigated polygamy except for the 2002 HIV/AIDS population-based household survey. The aim of this study was to profile socio-demographic and behavioural characteristics associated with women in polygamous relationships in South Africa using the 2002 survey data.

**Methods:**

The survey data were collected using a multi-stage stratified cluster randomised sampling design. Bivariate and multivariate logistic regression models were used to assess the relationship between polygamy, and selected socio-demographic and behavioural factors.

**Results:**

Of 1437 women who responded to the question on polygamy, 8.3% reported being in a polygamous marriage. Women in polygamous marriages were significantly less likely to have tertiary education [OR = 0.03(95% CI: 0.00–0.28), *p* = 0.003], to have money for food and clothes [OR = 0.12 (95% CI: 0.06–0.27), *p* < 0.001], to have a sexual partner five years younger [OR = 0.10 (95% CI: 0.01–0.94), *p* = 0.044] or sexual partner within 5 years older or younger [OR = 0.35 (95% CI: 0.13–0.991), *p* = 0.032]. They were also significantly more likely to have two or more sexual partners [OR = 20.42 (95% CI: 1.10–379.89), *p* = 0.043].

**Conclusion:**

The finding that polygamy is associated with uneducated and women of low economic means, who have relationships with older men and multiple sexual partners warrants further attention. Contemporary studies on polygamy are needed in South Africa.

## Background

Polygamy, which is defined as the practice of having more than one spouse, is a common, and widespread, socially as well as culturally accepted phenomenon in many African countries [1, 2]. The experiences of women in polygamous marriages vary according to the socio-cultural context [1]. Historically, many factors are thought to have perpetuated polygamy, and these include higher mortality rates of men, satisfaction of sexual desires, and the need to have as many children as desired [3]. Consequently, the practice of polygamy has been associated mainly with a patriarchal social system or societies in sub-Saharan Africa [3–5].

However, there is limited research and literature on the actual experiences of women in African polygamous families. Available evidence suggest that women in polygamous marriages generally experience varying degrees of emotional difficulties such as anger, jealousy, loneliness, unhappiness, emptiness and feeling of neglet [1, 5, 6]. Some studies suggest that the experiences and effects of polygamous relationships on women can be mediated by their socio-demographic background [5, 6]. Evidence shows that education, employment and place of residence were important determinants of being in polygamous marriages for women, and the effect varies depending on the context and setting [5, 6].

Polygamy may have negative effects and influences on women’s reproductive health [7–9]. These include barriers to conversations around family planning issues such as contraception use and child bearing. Moreover, women in polygamous marriages are at increased risk of acquiring sexually transmitted infections (STIs) including HIV, and being subjected to intimate partner violence. All these are attributed to gender-power differences in such relationships [7–9]. Since women in polygamous relationships tend to have less power they are more likely to suffer from sexual, emotional and psychological abuse [10].

Consequently, polygamy has been criticized [7–9]. However, polygamy continues to be practiced in much of Africa [7–9, 11]. In South Africa, polygamy has a long history in some cultures, and males with both middle and low socio economic background practice contemporary polygamy [11]. However, there is limited data on polygamy and its effect in South Africa. Unlike in other sub-Saharan countries [2–4], no nationwide survey has investigated polygamous relationships except for the 2002 national HIV prevalence, behavioural risks and mass media household survey. [12]. Given the risks such relationships pose to women in particular, it is pertinent to investigate the characteristics and behaviour of the female counterparts within polygamous relationships [3–6]. The aim of this study was to profile socio-demographic and behavioural characteristics associated with women in polygamous relationship using the 2002 national survey data.

## Methods

### Study data and sampling

The study used data from the population based nationally representative household survey of the 2002 South African HIV prevalence, Behavioural Risk and Mass Media Survey [12]. The target population for this study was all people living in households in South Africa excluding persons in special institutions (e.g. hospitals, military camps, old age homes, schools and university hostels). The sample size estimation was guided by the requirement for measuring change over time in order to detect a change in HIV prevalence in each of the main reporting domains at 5% level of significance, 80% power, two-sided test, and with a precision level of less than ±4%, and a design effect of 2. A total of sample size of 15,000 households / visiting points (VPs) was estimated for the survey based on these requirements.

A random sample of 15 VPs was selected using small units called enumerator areas (EAs) as defined by the 2001 population census from Statistics South Africa. One thousand EA’s were selected for inclusion in the study from a database of 86,000 EAs, yielding a total sample size of 15,000 households or VPs. The survey data were collected using multi-stage disproportionate, stratified sampling of residential households within EAs by province, race group and locality type (urban/rural and formal/informal). All people in all the selected households were initially listed, and eligible individuals randomly selected to each represent age groups 2–14 years, 15–24 years, 25 years and older.

Age-appropriate individual questionnaires including parent/guardian for minors were administered to consenting eligible individuals to solicit information that included demographic characteristics, media and communication on HIV, sexual behaviours and practices related to HIV, and marriage practices such as polygamy where applicable. Out of a total of 13,518 individuals who were selected and contacted for the survey, 9963 (73.7%) people agreed to be interviewed. The current analysis is based on the sub-sample of adult data (25 years and older) of women who responded to the polygamy question.

### Measures

The primary outcome variable is polygamy based on the question “does your husband have other wives” (yes = 1 and *n* = 0). Explanatory variables included socio-demographic variables such as age (15 to 24 years = 1, 25 to 49 years = 2, 50+ years = 3), race (Black African = 1 and other races = 2 i.e. White, Coloured, and Indians/Asians), educational level (no education = 1, primary = 2, secondary = 3, tertiary = 4), employment status (not employed = 1, employed = 2), household vulnerability indicator (Not enough money for basic things like food and clothes = 1, money for food and clothes but short of many other things = 2, have most of the important things but few luxury goods = 3), money for extra things such as holidays and luxury goods = 4, type of religion (Christianity = 1, other religion = 2), locality type (urban formal = 1, urban = 2 informal = 3, rural informal = 3 and rural formal = 4), and province- (Western Cape = 1, Eastern Cape = 2, Northern Cape = 3, Free State = 4, KwaZulu-Natal = 5, North West = 6, Gauteng = 7, Mpumalanga = 8, Limpopo = 9).

Sexual and other behavioural factors included age at early sexual debut (less than 15 years = 1 years, 15 years or more = 2), age mixing sexual partnerships (partner 5 years and older = 1, partner five years younger = 2, partner within 5 years = 3), number of partners in the last 12 months (one partner = 1, two or more sexual partners = 2), condom use at last sex (no = 1, yes = 2), self-perceived risk of HIV infection (no = 1, yes = 2), ever tested for HIV (no = 1, yes = 2), awareness of HIV status (positive = 1, negative = 2).

### Statistical analysis

Descriptive statistics (frequency distribution and percentages) were used to characterize socio-demographic and behavioural profiles of women in polygamous marriages. Chi-square tests were used to assess differences among categorical variables. Bivariate logistic regression models were fitted to assess the relationship between polygamy, socio-demographic and behavioural factors. Statistically significant variables were then entered into a multivariate logistic regression model to determine factors independently associated with women in a polygamous marriage. A *p*-value of ≤0.05 was considered significant in all statistical analysis. All data were analysed using statistical software STATA version 13.0 (Stata Corp, College Station, Texas, USA).

## Results

### Polygamy and characteristics of the study participants

**Fig. 1:**
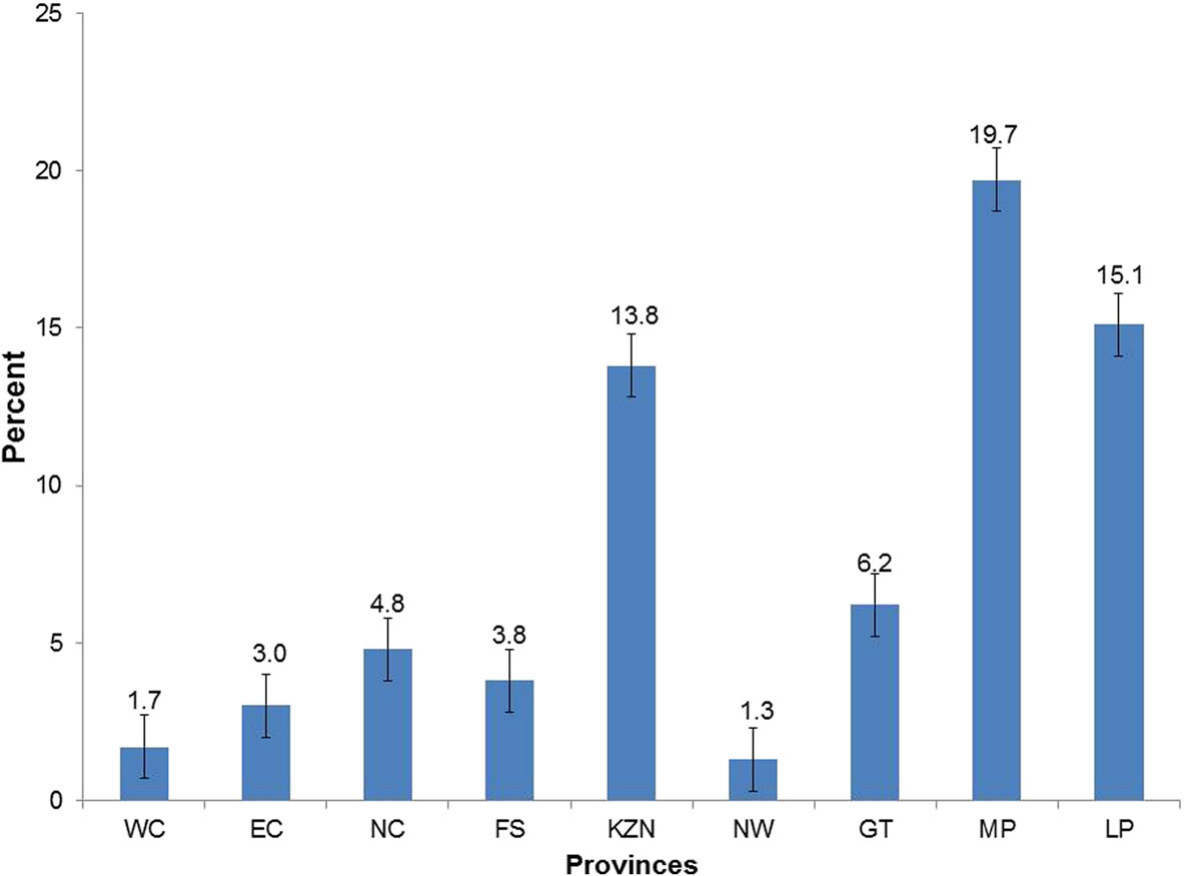
Proportion of women in polygamous marriages in the Western Cape (WC), Eastern Cape (EC), Northern Cape (NC), Free State (FS), KwaZulu-Natal (KZN), North West (NW), Gauteng (GT), Mpumalanga (MP) and Limpopo (LP) Provinces of South Africa

Of 1437 self-reported married women who responded to the question on polygamy, 8.3% (95% CI: 5.6–12) indicated that they were in a polygamous relationship. Figure 1 shows that polygamous marriages were common in Mpumalanga (19.7%), Limpopo (51.1%) and KwaZulu-Natal provinces (13.8%).

Table 1 shows that polygamous marriages were significantly more common among women aged 50 years and older (14.6%, *p* = 0.001), uneducated (22.7%, p = 0.001) and lacking enough money for basic things like food and clothes (14.2%, *p* = 0.005). Although non-significant it was also common among women who were Black African (10.7%), unemployed (8.9%) and who resided in tribal (13.2%), urban informal (12.0%) and rural formal (11.9%) areas. It was also common among those who indicated they were of Christian religion (7.8) and who believed that religion was not important at all (18.5%).

**Table 1:** Polygamous marriage among women by socio-demographic profile

Age (years)	Total^a^	%	95% CI	*p*-value
25 to 49	961	4.0	2.0–7.9	0.001
50+	476	14.6	9.3–22.2	
Race group				
Black African	750	10.7	7.2–15.5	0.053
Other	686	3.0	0.8–11.1	
Education level				
No education	208	22.7	13.9–34.8	< 0.001
Primary	611	7.2	4.4–11.3	
Secondary	456	2.6	0.7–9.5	
Tertiary	155	0.5	0.1–2.9	
Employment status				
Not Employed	960	8.9	5.8–13.3	0.465
Employed	442	5.9	2.1–15.7	
Household Vulnerability indicator				
Not enough money for basic things like food and clothes	521	14.2	9.3–21.1	0.005
Have money for food and clothes, short on many other things	485	6.3	2.6–14.6	
We have most of the important things, but few luxury goods	310	2.1	1.0–4.7	
Some money for extra things as holidays and luxury goods	104			
Type of religion				
Christian religion	1032	7.8	5.0–12.0	0.247
Other religion	303	3.9	1.2–11.6	
Importance of religion				
Not important at all	21	18.5	2.7–65.0	0.538
Slightly important	24	6.1	1.4–22.9	
Somewhat important	28	17.9	3.2–58.9	
Important	214	5.7	2.5–12.5	
Very important	1133	8.6	5.5–13.1	
Locality type				
Urban Formal	931	4.2	2.1–8.5	0.083
Urban Informal	109	12.0	3.9–31.3	
Rural informal	326	13.2	8.0–20.9	
Rural Formal	71	11.9	2.5–41.2	

^a^Subtotals do not add up to the overall total due to non-response and / or missing data

Table 2 shows that a significant majority of women in polygamous marriages had two or more sexual partners in the past twelve months (24.0%, *p* = 0.021), had sexual partners five years and older (7.7%, *p* = 0.003), had never tested for HIV (10.1%, p = 0.003), and were not aware of their HIV status (9.8%, *p* = 0.008).

**Table 2:** Polygamous marriages among women by behavioural profile

	Total^a^	%	95% CI	*p*-values
Number of sexual partners in the last 12 month?				
2+ partners	15	24.0	3.9–71.1	0.021
1 partner	1075	4.5	2.5–8.0	
Condom use last sex				
No	286	7.2	2.0–23.3	0.737
Yes	142	9.1	3.7–20.8	
Age mixing				
Partners five years and older	379	7.7	3.4–16.6	0.003
Partners five years younger	40	1.0	0.1–7.0	
Partners within 5 year	563	2.4	1.1–4.8	
Self-perceived risk of HIV infection				
No	1008	8.5	5.5–12.9	0.895
Yes	414	8.0	3.9–15.9	
Ever tested for HIV?				
No	1015	10.1	6.7–14.7	0.003
Yes	412	3.3	1.5–6.9	
Awareness of HIV status?				
No	1054	9.8	6.6–14.4	0.008
Yes	370	3.6	1.6–7.5	
HIV status				
Positive	110	8.9	3.4–21.4	0.985
Negative	1100	8.8	5.7–13.3	

^a^Subtotals do not add up to the overall total due to non-response and / or missing data

### Factors associated with polygamy

Table 3 shows bivariate models of factors associated with women involved in polygamous unions. Women were significantly more likely to be in a polygamous relationship if they were younger than 50 years old, were uneducated, and did not have enough money for basic things like food and clothes. Women in polygamouse relationships were also significantly more likely to have an older sexual  partner, multiple sexual partners and to never have tested for HIV and to be unaware of their HIV status.

**Table 3:** Bivariate models of factors associated with women in polygamous marriages

Variables	OR	95% CI		p-value
Age (years)				
25 to 49	1			
50+	0.25	0.10	0.59	0.002
Race groups				
Black African	1			
Other	0.26	0.06	1.12	0.070
Education level				
No education	1			
Primary	0.26	0.12	0.57	0.001
Secondary	0.09	0.02	0.41	0.002
Tertiary	0.02	0.00	0.11	< 0.001
Employment status				
Not employed	1			
Employed	0.65	0.20	2.10	0.468
Household vulnerability indicator				
Not enough money for basic things like food and clothes	1			
Have money for food and clothes, short on many other things	0.41	0.14	1.14	0.087
We have most of the important things, but few luxury goods	0.13	0.05	0.34	< 0.001
Some money for extra things as holidays and luxury goods				
Type of religion				
Christian religion	1			
Other religion	0.48	0.13	1.71	0.257
Number of sexual partners in the last 12 months				
One partner	1			
Two or more partners	6.66	1.05	42.08	0.044
Condom use last sex act				
No	1			
Yes	1.28	0.30	5.42	0.737
Age mixing				
Partner five years and older	1			
Partner five years younger	0.12	0.01	1.08	0.059
Partner within 5 years	0.29	0.12	0.71	0.007
HIV risk perception				
No	1			
Yes	0.94	0.40	2.24	0.895
Ever tested for HIV?				
No	1			
Yes	0.30	0.13	0.69	0.005
Awareness of HIV status?				
No	1			
Yes	0.34	0.15	0.78	0.011
HIV status				
Positive	1			
Negative	0.10	0.34	2.90	0.985

In the final multivariate  model (Table 4) women in polygamous marriages were significantly less likely to have tertiary education [OR = 0.03(95% CI:0.00–0.28), *p* = 0.003], to have money for food and clothes [OR = 0.12 (95% CI: 0.06–0.27), *p* < 0.001], to have sexual partner who was five years younger [OR = 0.10 (95% CI: 0.01–0.94), *p* = 0.044], and sexual partner within 5 years older or younger than their age [OR = 0.35 (95CI: 0.13–0.91), *p* = 0.032]. On the other hand they were significantly more likely to have two or more sexual partners [OR = 20.42 (95% CI: 1.10–379.89), *p* = 0.043].

**Table 4:** Multivariate model of factors independently associated with women in polygamous marriages

Variables	OR	95% CI		p-values
Education level				
No education	1			
Primary	0.66	0.17	2.53	0.544
Secondary	0.68	0.13	3.60	0.650
Tertiary	0.03	0.00	0.28	0.003
Household vulnerability indicators				
Not enough money for basic things like food and clothes	1			
Have money for food and clothes, short on many other things	0.12	0.06	0.27	< 0.001
We have most of the important things, but few luxury goods	0.22	0.04	1.25	0.088
Locality Type				
Urban Formal	1			
Urban Informal	0.31	0.05	1.95	0.213
Tribal	0.42	0.08	2.29	0.314
Rural Formal	2.17	0.40	11.71	0.367
Number of sexual partners in the last 12 months				
One partner	1			
Two or more partners	20.42	1.10	379.89	0.043
Age mixing				
Partner five years and older	1			
Partner five years younger	0.10	0.01	0.94	0.044
Partner within 5 years older or younger	0.35	0.13	0.91	0.032

## Discussion

This analysis profiled factors associated with self-reported polygamy among women using data from the 2002 nationally representative household survey. The relatively high prevalence of women reporting polygamous marriage in Mpumalanga province followed by Limpopo and KwaZulu-Natal provinces probably reflects the cultural contexts in these provinces. For example, the practise of polygamy is predominant among the Shangaan, Swati and Zulu tribes in South Africa [12, 13], which are found in these provinces.

The findings showed that woman’s lack of education and lack of economic empowerment play predominant roles in polygamous relationships. Elsewhere in Africa evidence shows that involvement in polygamous marriage declines with increase in women’s education from secondary to higher level [3, 9]. This has been attributed to the fact that woman who are more educated are more likely to be economically independent and more likely to have power in relationships and hence are less likely to be in polygamous marriages [3, 14].

The findings also revealed that women in polygamous marriages were more likely to reside in financially vulnerable households with less money for food and short on many other things. This probably reflects the economic context of polygamy for the study population, which invariably transfers heavy economic burden to families of polygamous marriages where limited resources need to be stretched. Evidence shows that regardless of cultural differences the practice of polygamy impacts women’s livelihood in complex ways rendering them socially, economically and psychologically vulnerable [15].

Additionally the findings revealed that women in polygamous marriages have older partners. This is in line with evidence which suggests that mostly older males engage in polygamy rather than younger men [14]. Generally, this confirms observed patterns in most African communities where girls became brides shortly after puberty, while men get married at a more advanced age [16]. Typically, in polygamous marriages men often seek younger wives to satisfy their sexual needs. This perception is socially constructed around the assumption of men’s biologically determined greater sexual needs, which requires them to have several and often younger female partners to satisfy those needs [13].

The findings also showed that women in polygamous relationships were more likely to have that multiple sexual partners. It has been suggested that women in polygamous relationship often have clandestine affairs with other men [13]. This may be associated with psychological stress to due to lack of marital partner commitment and / or partner attachment and sexual satisfaction [17, 18]. The main point is that the women in such relationship are often unhappy with their marriage life but are limited by social and economic conditions in which they find themselves. For these reasons, women are more likely to find ways to manoeuvre and strive for wellbeing within the confines of an unhappy marriage [19].

### Limitations

The results have several limitations and should be carefully interpreted. The cross-sectional study design is limited to determining factors associated with polygamy and makes it difficult to infer causality. There may also be other unmeasured confounding factors, which have an effect on the association between polygamy and selected factors. Furthermore, the data collected were self-reports, which may be prone to social desirability bias. The analysis may have also been affected by non-response and / or missing data. The other limitation was the relatively limited number of women who responded to the question on polygamous marriages. The retrospective nature of the analysis is reflective rather than prospective. This means that generalization to the current population of women in polygamous relationships  could not be made. Nevertheless, the results provide a basis for future research in this field in South Africa.

## Conclusion

Polygamy is a social phenomenon that has existed for millennia and continues to transform itself in sub-Saharan. The retrospective data presented in the current study revealed evidence of low levels of education, marriage to older male partners, and involvement in multiple sexual partnerships among women in polygamous marriages. More contemporary studies are needed on the impact of polygamy on women in light of increasing levels of modernization including changes in the socio-economic and demographic features of the South African society.

## Abbreviations

AIDS: Acquired immune deficiency syndrome
CI: Confidence intervals
DHHS: Department of Health and Human Services
DHS: Demography and health survey
FWA: Federal Wide Assurance
HIV: Human Immunodeficiency Virus
OR: Odds ratio
